# Supplementary material for: Analysis of Phytohormone Signal Transduction in Sophora alopecuroides under Salt Stress
Source: Int J Mol Sci. 2021 Jul 7;22(14):7313. doi: 10.3390/ijms22147313 (PMC8304577; doi:10.3390/ijms22147313)
Supplement: Supplementary file 1 [file ijms-22-07313-s001.zip › Supplementary Materials.pdf]

Table S1 QRT-PCR primers

Gene	Forward primer (5' to 3')	Reverse primer (5' to 3')
SaAUX1	TTCATTGTGGTGTGGGTTTT	TGTGGTGGTGTAGGAGGCTT
SaARF	TGCCGAAAGAGACTCAAAGC	GGGGTCAAAAAGCCAAATAA
SaAHP	TAGTGTGATACGAGGCAGAA	AAGAGCAAGACAGAGTGGAC
SaARR	ATCTTTACATACTTTTTCAG	AACAGTTTTTTTCTCTCCTT
SaCYP90B1	CTTGGAAATGTAGTGAGGTT	ATTGAAGTGTTGAGGTTGGT
SaCYP85A1	GATCCACTAACATTCAACCC	ATTTCTGCTATTCCCAACTC
SaCYCD3	TGTTGGTTGTCTTCCTTCTA	CTCATCTACTTTCTCCTTGT
SaGA2ox	CATCAAAACAACAACCACAT	TTCTTCACAGGCATTCACTA
SaGID1	TCCTTCTTCATCCATTGTTT	TGGTCTCTATCTTCCCCTTC
SaDELLA	AACAAGAAGCGAACCACAAC	AAACCTCCGACATAAGCAAA
SaGID2	CAGGATGAGAGGCTTTGGGA	AAGGGAATGGAGACGGCGGA
SaETR	AGTGATAGCGTTGAGGGCAG	GTGTAATTGAAACCGAAGAA
SaJAR1	CTAAGGCAATAAATAGCACA	CAGAATAAAAACCAGAAAAA
SaJAZ	TATCATCCCTAGTACCGGCA	CCTATCCTTTCTCTTCTCCA
SaPYL	AGCCTCTCCCCTTCACCAAA	CGCCGGACGACGGACCACAC
SaPP2C	GTAACATCGGTTTGTGGTAG	CGAAGTAGTGGAAGTTTGGA
SaABF	TCTTTAACTACATTTCTTCG	CATTGTCTTTTACCTGCCCA
SaTGA	CTGTGATTGAAATAGGGAAG	AAAATACAAAGGGAGTGGGG
SaEBF1/2	GGAGGAGTGCAACAGGGTTA	CGCAAGGAGAGAGCATAGAT
Actin	GTCCTTTCAGGAGGTACAACC	CCACATCTGCTGGAAGGTGC

Gene name	Gene ID	FPKM-value				
		0h	4h	24h	48h	72h
gapN						
SagapN	Cluster-68696.30093	18.33	30.62	43.28	39.37	64.11
GAPDH						
SaGAPDH-1	Cluster-10641.0	0.00	0.06	0.06	0.00	4.73
SaGAPDH-2	Cluster-14337.0	0.00	0.14	0.05	0.13	4.00
SaGAPDH-3	Cluster-68696.45462	79.24	100.30	150.80	134.27	409.44
SaGAPDH-4	Cluster-68696.45464	48.54	52.91	84.62	81.01	369.77
SaGAPDH-5	Cluster-16722.0	0.06	0.00	0.08	0.00	2.83
SaGAPDH-6	Cluster-68696.18254	1.66	0.10	0.34	0.05	0.08
SaGAPDH-7	Cluster-68696.20112	2.18	1.59	1.78	1.27	0.36
PDHA						
SaPDHA	Cluster-68696.38287	50.53	37.71	57.99	50.64	16.57
PDHB						
SaPDHB	Cluster-68696.46074	66.88	53.73	75.25	64.07	28.23
DLD						
SaDLD-1	Cluster-68696.41241	33.36	38.82	46.91	32.59	56.93
SaDLD-2	Cluster-68696.48772	33.63	20.28	39.31	33.61	8.00
DLAT						
SaDLAT-1	Cluster-68696.47310	21.96	13.35	18.84	18.67	7.83
SaDLAT-2	Cluster-68696.49308	36.56	30.36	43.00	39.12	11.28
PK						
SaPK-1	Cluster-68696.25346	0.49	1.31	1.29	0.38	1.92
SaPK-2	Cluster-68696.35450	62.79	70.65	75.01	85.15	157.37
SaPK-3	Cluster-68696.41515	4.37	3.47	3.86	4.48	1.62
SaPK-4	Cluster-68696.32470	2.82	1.91	2.45	2.65	0.46
SaPK-5	Cluster-68696.32471	19.47	13.61	18.05	17.87	6.19
SaPK-6	Cluster-68696.28417	8.05	4.49	4.39	4.47	0.68
PGK						
SaPGK	Cluster-68696.38653	142.78	112.84	172.20	182.98	324.48
PDC						
SaPDC-1	Cluster-47957.0	0.09	0.32	3.60	0.27	2.93
SaPDC-2	Cluster-68696.39182	34.72	20.33	21.72	22.51	73.10
pckA						
SapckA-1	Cluster-68696.64166	0.78	2.33	15.04	0.76	7.56
SapckA-2	Cluster-68696.64167	1.07	2.66	10.77	0.64	7.76
SapckA-3	Cluster-68696.64168	0.09	0.14	0.50	0.06	5.67
gpmB						
SagpmB	Cluster-68696.21005	1.54	3.71	2.35	2.32	0.81
trpB						
SatrpB	Cluster-68696.38582	9.50	15.53	16.40	19.58	18.88
HMGCR						
SaHMGCR	Cluster-68696.28853	12.53	30.16	17.82	15.40	5.83
atoB						
SaatoB-1	Cluster-68696.40709	47.01	48.96	41.05	43.67	14.74
SaatoB-2	Cluster-68696.46632	12.73	20.01	17.20	16.04	27.53
MVK						
SaMVK	Cluster-68696.33284	24.77	20.84	20.37	22.16	8.55
MVD						
SaMVD	Cluster-68696.34533	10.33	21.80	12.61	9.97	4.04
dxs						
Sadxs-1	Cluster-68696.17020	0.35	2.16	2.31	0.23	3.77
Sadxs-2	Cluster-68696.37411	14.72	17.85	19.30	13.35	36.91
gcpE						
SagcpE-1	Cluster-68696.11737	3.03	8.66	11.09	4.25	14.04
SagcpE-2	Cluster-68696.36523	4.15	8.78	10.01	6.84	12.21
SagcpE-3	Cluster-68696.39119	6.72	10.82	12.02	8.42	23.06
SagcpE-4	Cluster-68696.39120	66.98	102.08	130.78	94.46	167.80
ispH						
SaispH	Cluster-68696.38530	23.07	26.61	33.17	25.65	40.29
ispS						
SaIspS-1	Cluster-68696.49140	27.33	10.94	15.71	14.16	0.86
SaIspS-2	Cluster-68696.53918	5.29	29.06	14.37	12.14	4.85
GGPS						
SaGGPS-1	Cluster-68696.33893	16.28	27.56	22.04	19.53	34.06
SaGGPS-2	Cluster-68696.55675	5.77	4.92	4.02	2.69	1.16
TGL4						
SaTGL4	Cluster-68980.0	0.04	0.08	0.44	0.04	0.39
LOX2S						
SaLOX2S-1	Cluster-68696.21694	5.76	3.25	0.91	2.77	0.14
SaLOX2S-2	Cluster-68696.46534	32.49	48.63	51.18	39.67	83.01
SaLOX2S-3	Cluster-68696.27488	3.15	2.37	5.29	2.22	8.84

Low High

Gene name	Gene ID	FPKM-value				
		0h	4h	24h	48h	72h
AOC						
SaAOC-1	Cluster-68696.37359	26.85	61.45	28.95	25.47	13.51
SaAOC-2	Cluster-68696.4115	3.19	10.58	4.69	6.17	3.57
SaAOC-3	Cluster-68696.51674	10.68	52.21	22.13	23.00	16.42
SaAOC-4	Cluster-68696.4551	1.33	4.43	2.01	3.63	3.63
OPR						
SaOPR-1	Cluster-68696.3878	0.02	0.55	0.55	0.17	2.65
SaOPR-2	Cluster-68696.52034	6.44	16.32	16.13	10.52	34.10
OPCL1						
SaOPCL1	Cluster-48419.0	0.47	0.12	0.12	0.49	0.00
ACOX						
SaACOX-1	Cluster-36762.0	0.00	0.00	0.00	0.03	1.93
SaACOX-2	Cluster-68696.40264	13.46	25.83	28.84	21.82	43.28
SaACOX-3	Cluster-68696.40264	13.46	25.83	28.84	21.82	43.28
SaACOX-4	Cluster-68696.34465	9.54	20.80	15.30	12.21	14.97
ACAA1						
SaACAA1-1	Cluster-40047.0	0.00	0.09	0.00	0.00	1.66
SaACAA1-2	Cluster-56006.0	0.52	0.68	1.54	0.30	2.20
SaACAA1-3	Cluster-31831.0	0.00	0.00	0.02	0.00	7.36
SaACAA1-4	Cluster-7233.0	0.00	0.00	0.03	0.02	3.20
FDFT1						
SaFDFT1-1	Cluster-47691.0	4.52	3.52	2.55	2.93	0.30
SaFDFT1-2	Cluster-68696.50280	9.75	13.13	8.37	8.00	2.62
SMT1						
SaSMT1	Cluster-68696.51166	7.22	6.89	7.05	6.92	14.11
EBP						
SaEBP-1	Cluster-68696.28730	1.88	0.94	1.16	1.34	0.30
SaEBP-2	Cluster-68696.28731	5.38	2.56	2.82	2.61	0.16
SMO2						
SaSMO2	Cluster-68696.38971	6.35	5.84	3.99	5.30	1.72
DWF1						
SaDWF1	Cluster-68696.36002	143.83	84.98	87.81	107.07	34.12
CHM						
SaCHM	Cluster-68696.48911	19.20	25.07	24.92	20.70	34.39
TAT						
SaTAT	Cluster-68696.47966	161.70	45.99	20.38	51.44	11.35
ADT						
SaADT	Cluster-68696.28996	30.79	26.27	45.23	32.63	63.91
cysE						
SacysE	Cluster-68696.44235	15.59	40.61	39.04	23.91	81.09
CTH						
SaCTH-1	Cluster-33459.0	0.04	0.00	0.00	0.08	1.93
SaCTH-2	Cluster-33745.0	0.00	0.04	0.03	0.00	1.84
metC						
SametC	Cluster-68696.35644	15.44	9.99	11.06	13.03	5.82
BHMT2						
SaBHMT2-1	Cluster-68696.44207	41.85	33.25	34.64	37.65	17.59
SaBHMT2-2	Cluster-68696.46569	1.31	5.38	8.95	5.91	16.47
SaBHMT2-3	Cluster-68696.46570	0.32	1.18	2.62	1.09	4.07
metK						
SametK-1	Cluster-68696.26193	23.29	13.26	8.77	8.53	0.97
SametK-2	Cluster-68696.26193	23.29	13.26	8.77	8.53	0.97
SametK-3	Cluster-44488.0	0.02	0.00	0.07	0.00	1.97
SametK-4	Cluster-9266.0	0.00	0.12	0.21	0.00	1.55
SametK-5	Cluster-68696.37979	384.28	508.16	557.54	435.08	901.27
SametK-6	Cluster-68696.13913	0.00	0.04	0.19	0.02	2.99
SametK-7	Cluster-68696.39020	434.85	646.36	853.39	643.84	998.92
SametK-8	Cluster-68696.15105	0.00	0.02	0.07	0.34	12.63
SametK-9	Cluster-68696.43308	4.24	3.35	3.93	4.06	8.38
SametK-10	Cluster-33191.0	0.00	0.00	0.00	0.05	2.06
SametK-11	Cluster-35657.0	0.00	0.00	0.00	0.02	2.27
SametK-12	Cluster-53330.0	0.04	0.04	0.02	0.08	2.32
NCED						
SaNCED	Cluster-68696.20426	1.53	255.14	37.13	9.02	16.73
ZED						
SaZED-1	Cluster-68696.51755	1.29	3.98	4.45	3.19	2.52
SaZED-2	Cluster-68696.51759	4.13	10.04	7.58	7.05	6.04
CrtZ						
SaCrtZ-1	Cluster-68696.57023	6.76	172.87	64.95	16.11	64.25
SaCrtZ-2	Cluster-68696.57022	2.40	67.94	20.85	5.74	17.27
ADT						
SaADT	Cluster-68696.28996	30.79	26.27	45.23	32.63	63.91

Low High

Figure S1 Heat map of Phytohormone biosynthetic pathway-related gene expression. Values are Average FPKM value of each sample in each group.

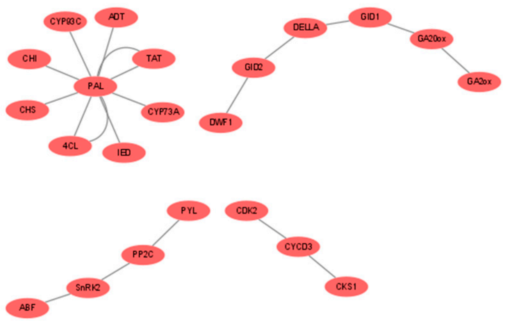

Figure S2 Protein interaction network analysis

Video S1 The phenotypic changes of *S. alopecuroides* under salt stress
